# Controversies in the prevention and control of antimicrobial drug resistance.

**DOI:** 10.3201/eid0403.980338

**Published:** 1998

**Authors:** D Bell

**Affiliations:** Centers for Disease Control and Prevention, Atlanta, Georgia, USA.


### Controversies in the Prevention and Control of Antimicrobial Drug Resistance

**David Bell**
Centers for Disease Control and Prevention, Atlanta, Georgia, USA

### In Hospitals

William Jarvis, Centers for Disease Control and Prevention (CDC), discussed antimicrobial resistance related to hospitalization. Two major factors contribute to the emergence and spread of antimicrobial resistance in hospitals: a high rate of antimicrobial drug use and inadequate infection control practices. Much antimicrobial drug use in hospitals is inappropriate (e.g., the use of vancomycin to treat a staphylococcal infection susceptible to methicillin, or the continuation of perioperative prophylaxis beyond 24 to 48 hours). Educational efforts on antimicrobial drug use in hospitals have had mixed success. More aggressive and controversial approaches to improve the use of these drugs have been proposed; for example, excluding certain drugs (such as vancomycin) from the routine reporting of susceptibility results; monitoring antimicrobial use with feedback to physicians concerning inappropriate use; antibiotic-use audits targeting problem areas (e.g., no diagnostic test done, more than four drugs used during one hospitalization, use for more than 3 weeks continuously); regulating drug promotion; requiring justifications for use; using computer-generated stop orders; and developing formularies, restrictions, and protocols by a multidisciplinary team.

### In Communities

Keith Klugman, South African Institute of Medical Research, spoke on community-acquired infections, focusing on respiratory pathogens. One controversial area concerns the extent to which drug resistance identified in the microbiology laboratory correlates with clinical failure. Since clinical trial data are frequently unavailable, assessment of drug efficacy is often based on pharmacodynamics; i.e., a drug is believed efficacious if its concentration at the site of infection exceeds the organism's MIC. Otitis media and meningitis studies support this approach. In Pakistan, laboratory data indicate that 78% of pneumococci are resistant to cotrimoxazole, yet the clinical treatment failure rate is only 15%. The reasons for this discrepancy are unknown, but the issue is important because alternative drugs are more expensive. Standardization of laboratory methods and appropriate surveillance methods are essential.

Another controversial area involves antibiotic use and how to improve it. For many infections, the optimal dose and duration of therapy are unknown. Antibiotics are often prescribed inappropriately because physicians are uncertain when antibiotics are indicated and patients demand them; educating both of these groups is a challenge. Better diagnostics to reduce empiric therapy would be helpful. Other areas of uncertainty include the use of vaccines to decrease colonization and infection with resistant organisms and the extent to which antibiotics given for a specific indication might lead to resistance in different organisms.

### In Veterinary Medicine

Klaus Stoehr, World Health Organization (WHO), addressed controversies related to use of antibiotics (preventive, therapeutic, growth-promoting) in food animals. Some antibiotic use contributes to the pool of resistant human pathogens. Both medical and nonmedical uses of antibiotics should be reduced. More scientific data are needed to address issues related to antibiotic use in food animals, including elucidating the human health impact, e.g., the percentage of resistance genes or resistant organisms originating in animals and the extent to which therapy of zoonotic bacterial infections in humans has been compromised because of resistance. The economic benefits of subtherapeutic antimicrobial use for growth promotion are also controversial; one study estimates that production costs without such use would increase by up to 8%, but recent experience in Sweden indicates that meat produced without growth promotants can be priced competitively. A WHO meeting in 1997 on the medical impact of antibiotic use in livestock production recommended antimicrobial resistance monitoring and prudent use of antibiotics in food animals.

### In Developing Countries

Antonio C. Pignatari, Escola Paulista da Medicina, São Paulo, Brazil, discussed antibiotic resistance issues in developing countries. More than two thirds of the world's population lives in developing countries, where the contrast between wealth and poverty is extreme. Infectious diseases represent the main public health problem. Because of inadequate resources for surveillance, control, and treatment, antimicrobial-resistant infections have become a major problem with serious implications for the health system and the economy. The main problems with drug resistance are seen in the treatment of diarrheal diseases, sexually transmitted diseases, pneumococcal infections, tuberculosis, nosocomial infections, and malaria. Restrictive antimicrobial use policies (which are controversial) can be effective in the hospital but are difficult to implement in the community. In many areas, the availability of medical care is limited; thus, laws requiring a physician's prescription for antibiotics are difficult to enforce. Pharmacies provide an important service in dispensing medications, yet most developing country pharmacists have limited training. The use of antimicrobial drugs in food animals is also a problem in developing countries, and no controls are in place to address it. Control of antimicrobial resistance and emerging infections in developing countries cannot be achieved without addressing closely related social and economic issues.

### In Clinical Laboratories

Fred Tenover, CDC, addressed antibiotic resistance issues in the microbiology laboratory. Laboratorians must move from susceptibility testing to finding resistance. A common misconception is that new resistance mechanisms are easily identified because they result in high MICs and low zone sizes. However, many new resistance mechanisms lead to MICs close to the breakpoint for resistance. More sensitive screening tests are being introduced to detect resistance, but they cannot replace MICs; therefore, laboratory workload is increasing in an era of downsizing.

Several "drug-bug" combinations are problematic and require new approaches. For detecting nonsusceptibility (intermediate or full resistance) of staphylococci to vancomycin, the traditional method of disk diffusion testing is not reliable. Acceptable methods include the Brain Heart Infusion vancomycin agar screening test developed for enterococci or broth microdilution tests held 24 hours. For pneumococci, testing for susceptibility to both penicillin and extended spectrum cephalosporins is important because resistance to these drugs is becoming more common. For invasive isolates where the need to detect resistance is urgent, the oxacillin screening test should not be used; MIC methods should be used directly. For gram-negative bacilli, traditional methods to detect resistance to extended spectrum beta-lactam drugs are inadequate, although the latest National Committee for Clinical Laboratory Standards guidelines present effective screening tests. Finally, sensitivity must be determined for clinically important isolates treated with fluoroquinolones, since selective pressure for resistance is increasing as a result of widespread use.

